# Civil Malpractice

**Published:** 1873-03

**Authors:** M. A. McClelland

**Affiliations:** Knoxville, Illinois


A MONTHLY RECORD OF

*Medicine, Surgery and the Collateral Sciences.*

EDITED BY J. ADAMS ALLEN, M.D., LL.D.; AND WALTER HAY, M.D.

---

VOL. XXX. — MARCH, 1873. — NO. 3.

---

### Original Communications.

ARTICLE I.—*Civil Malpractice. A Report presented to the Military Tract Medical Society, at its Fifteenth Semi-Annual Meeting, Jan.* 14, 1873. By M. A. MCCLELLAND, M.D., Knoxville, Illinois.

(Continued from page 97.)

To emphasize the danger attending the use of the "initial" bandage, the following case is in point:

*Steele* v. *Newton:* Superior Court of Cincinnati, Nov. Term, 1856.

This was an action to recover damages for improper treatment of a fracture of the lower end of humerus. Damages laid at $3,000. The defendant was an "Eclectic" practitioner of Cincinnati; and the Editor of a work entitled "Symes' Principles and Practice of Surgery," from which this case is quoted. "When stripped for inspection, it was found that motion of the elbow-joint was perfect. There was a visible decrease in the muscles of the forearm and partial contraction of the fingers. He stated that he sometimes scratched his hand so as to make it bleed without being aware of the injury. The contraction of the fingers was not more than is customary with those who walk with the hands swinging by the side."

PROF. R. D. MUSSEY, testified, "that he found the arm withered, and the general sensibility much impaired—results arising from diminished innervation. He inferred that the blood-vessels had been too much constricted by the bandages, thus obstructing the proper circulation of the blood in the limb. These results might have been induced by injury to the median nerve, but thinks the circulation was obstructed, and in consequence the blisters appeared. Fractures in the lower end of the humerus are very difficult to treat, and for which there is a variety of plans. He should have kept the forearm flexed on the upper-arm, at a right angle, and have been careful not to dress it too tightly. He should use some sort of a splint, though he could not at present specify the exact kind. He was of opinion that, if a fracture had existed in the case, it was probably an oblique fracture including the internal condyle. The elbow-joint is now in good condition, having its natural motion in all directions. He has not, however, seen similar results from such a fracture. The ulnar nerve, as well as the median, may have been injured."

*Cross-examined.*—He was not positive as to the direction of the fracture, nor was it always easy to judge so far as to form a positive opinion. All fractures of the os humeri, running into the elbow-joint, are difficult of cure. He was of opinion that a permanent injury, or a degree of deformity, is sustained in a majority of cases. The vesications in this case may be accounted for by the injury done to the nerves, but thinks that tight bandages are oftener the cause of such results. He has seen cases similar to this—had seen a case some years ago, where the forearm mortified in consequence of having been too tightly bandaged. Thinks that in this case the dressing may have been too tight. He was of the opinion that partial paralysis might be induced by too tight dressing, without gangrene necessarily ensuing. Erysipelas may follow such injuries, exhibiting itself in from twenty-four to seventy-two hours.

PROF. JESSE JUDKINS, testified, that wasting or withering may be a result of muscular changes or of nervous sympathy. If there had been a fracture of the arm, the reparation had been most complete. Was of the opinion that the inflammation had been very intense. The capillary circulation may have been arrested by too tight bandages, the result of which would have been inflammation, erysipelas and gangrene. Injuries of the elbow-joint are always attended with complications, the nature of which *cannot* be always easily determined. The erysipelas complained of was, in all probability, induced by too tight bandages. The surgeon should see such a patient at intervals ranging from two days to one week.

*Cross-examined.*—Does not think that the injury of the median nerve alone caused the difficulty here presented. Thinks the bandages might have caused the erysipelas. Bandages cannot be put on tight enough to paralyze, without producing gangrene. He thought that a majority of fractures of the elbow-joint were completely cured. When elbow-joint fractures are complicated, the majority were not cured completely.

Prof. T. Wood, testified, that he thinks present condition of the arm is the result of bandages too tightly applied. Could not discover evidence of there ever having been a fracture. Never met with such a result as this, except it had arisen from bandages too tightly applied. The median nerve did not supply all the fingers. He thought that the purple color of the hand, as testified to by plaintiff, arose from compression; the blistering being one of the first results of such compression. Paralysis may be induced without gangrene. No injuries of the elbow-joint are so completely cured as to leave no trace of them. Had known wasting of the arm to occur where the bone had sustained no injury.

Prof. G. C. Blackman, testified, that he had examined boy's arm and had heard his statement, but from those sources had formed no positive opinion. The paralysis may be the result of the shock sustained at the time of the accident, or it may be the result of too tight bandaging, or from having retained the arm too long in one position. The blisters common to such fractures may follow in less than twenty-four hours where there is no dressing, and may be the result of the violence done to the soft parts at the time of injury. If erysipelas was, at the time, epidemic, it would almost certainly follow as one of the phases of such a case as the one under consideration, or even a less injury. He had no positive proof that the arm had been fractured, but he thought there had been a fracture of the humerus, involving the inner condyle and injuring the ulnar nerve. These are bad fractures, and their true character is difficult to detect. He thought that the ends of the bones should have been put together immediately, inflammation or not. The bandages in such oblique fractures must be tied rather tightly, and the surgeon may use either the wooden or paste-board splints. Had known mortification occur in twenty-four hours from tight bandages. The blisters may have been the result of such bandages.

*Cross-examined.*—Could not say that the bandages in this boy's case had been too tight. In such fractures it is very common to have impaired motion. The experience of the oldest and ablest surgeons in both Europe and America show this to be the case. Prof. Hamilton shows that a majority of such cases are attended with permanent injury of some sort, and all authors on the subject testify that an impaired condition results in a large majority if not in all cases. Conditions might arise which would cause him to remove the bandages entirely, as severe pain, inflammation, etc. He considered the repair in this case very perfect, and he had seen greater paralysis arise from less injuries.

Prof. R. S. Newton, the defendant in the case, testified, that he was a Professor in the Eclectic Medical Institute and Surgeon to Newton's Clinic Institute. On the day set forth in the declaration saw the boy, Steele, with his arm broken. Humerus broken off obliquely, the end of the bone driven down into the hollow of the arm. Boy suffering very much. Could distinctly feel the end of the bone. Had experienced but little difficulty in setting it, but it was not so easy to keep it in proper position, there being a constant tendency to slip down; hence, to prevent this, the bandage had to be applied firmly. The arm was much swollen at the joint, and all the blood vessels of the arm seemed to be engorged, though not more than an hour had elapsed since the accident. He first drew the point of the bones together and then bandaged the arm from the hand up, afterwards applying the splints, leaving the arm flexed at right angles. In this case more pressure was needed than in simple transverse fracture, on account of its obliquity. Visited boy next morning with Dr. Freeman. Boy complained of arm hurting him; on removal of dressing found arm blistered, part of the arm and hand was of a darkish red, but there were no indications of gangrene. He said to Mrs. Steele that it was a bad fracture, and that he would hold the arm till she could get her family physician or another surgeon, unless she would assume the responsibility of the case. She told him to go on and do the best he could. He had explained to her the dangers of such a fracture. After puncturing several of the blisters, he reapplied the bandages, and then put on two splints; one on the inside and one on the outside of the arm. The bandage extended from the ends of the fingers above the fracture, the hand being bandaged straight. One of the splints was removed in a few days. When the long wooden joint splint was off the inside, he had an elastic splint on in its place, and a long wooden one on the outside. He treated the blisters with water dressings and continued the splints four weeks or longer. The arm became offensive about one week after the accident. Had the boy under his care about seven months. Had treated the case as a charity patient, never having made a charge against Mrs. Steele on his books.

Prof. A. H. Baker, testified, that the erysipelas spoken of was a result of inflammation, and this had been induced by the injury done to the soft tissues. The indications of a fracture in such a young patient may be so completely removed in eighteen months or two years that no mortal man can detect it. The first bandage he considered most important. The practice of Dr. Newton in this case was proper, and such as would have been followed by any judicious surgeon.

Prof. Z. Freeman, testified, that he saw patient next morning after the accident. The hand and arm were purple. There were blisters on the hand and arm. The bandage was not too tight. It was an oblique fracture above condyles. The boy's mother declined calling in any other physician, and both she and the boy requested Dr. Newton to go on and treat the case. The blisters in this case were, in his opinion, the result of the attending erysipelas.

The Hon. O. M. Spencer, in his charge to the jury, said: "It is due to the professional man, who has treated a case in other respects fairly and attentively, that a candid and favorable consideration should be given to the judgment which he may form of his duty during the progress of that case; otherwise no physician or surgeon would dare to undertake, or be safe in the performance of his undertaking. In a case otherwise doubtful, this consideration alone should preponderate in his favor.

"Upon the whole, gentlemen, we declare that to entitle the plaintiff to a verdict you must be satisfied from this evidence, that the injury of which he complains was not the natural result of the original accident, but was distinctly traceable to the mode of treatment pursued by the defendant; in the adoption and continuance of which he did not apply that skill and care which men of ordinary intelligence and prudence, as physicians and surgeons, would have applied. Should this be your conviction, the plaintiff must recover such damages as will compensate him for the injury sustained, not exceeding the amount claimed. Should it be otherwise, or should you not be able to trace the cause of this injury, or your minds be unable to decide from the evidence whether the defendant has been in fault, according to the rule stated, your verdict should be for the defendant."

*Verdict*:—"We the jury, find the issue joined for the defendant."

The difficulties the professional witnesses had here to contend with were very great. The conclusion most surgeons would come to in such a case, would be that it was probably the result of absurd bandaging. Certainly no surgeon, except one who had seen all "indications of a fracture in the elbow-joint removed in eighteen months or two years," would thereafter consider "the first bandage" a *sine qua non.*

The only case that came before the Knox County Court of which records have been preserved, is that of *Sherman* v. *Bunce and Morse.* I am induced to present it in this connection, on account of the very just, as I view it, instructions given in behalf of the defendants. It exhibits also the character of those persons from whom we mostly expect suits of this character.

Knox Circuit Court, April Term, A. D. 1858—Action on the Case.

PLAINTIFF'S DECLARATION: That he suffered a fracture of right leg. That defendants held themselves out as physicians and surgeons; were employed by plaintiff to treat the fracture, and when the fractured bone should have begun to heal, they, contrary to expressed wish of plaintiff, stretched the broken bone apart, causing much pain, and thus retarded recovery and rendered plaintiff a cripple for a long time, to wit, one year and six months; damages laid at $6,000; shortening about one inch. Doubtless much less, as these kind of people amplify to the utmost possible limit.

The plaintiff swears he is too poor to prosecute said suit, and asks to prosecute said suit without cost. (Affidavit of Plaintiff.)

The instructions of the court (THOMPSON, J.) were as follows:

1. In this case the plaintiff is bound to prove by a preponderance of evidence the material allegation in some one count in his declaration, or he is not entitled to recover at all in this action.

2. Unless the jury believe, from the evidence, that the defendants did not use reasonable skill as surgeons in the treatment of the plaintiff's limb, or that they did not give reasonable attendance to the cure of said limb; and unless the jury further believe that on account of such want of skill or attendance the plaintiff had a worse limb than might reasonably be expected under the circumstances, as proved, then they should find the defendants not guilty.

3. If the jury believe, from the evidence, that under the treatment of the defendants, if such treatment was reasonably skillful, the plaintiff obtained as good a leg as could be reasonably expected under the circumstances, as detailed in evidence, of the condition of plaintiff, and such manner of treatment, they should find the defendants not guilty.

4. The defendants are only bound to use reasonable skill and to give the usual reasonable attendance to their patient in such cases; if they do this and a perfect cure is not obtained, they do not thereby become liable to damages to their patient.

5. If the jury believe, from the evidence, that a perfect cure or adjustment and union of the bones was prevented or retarded by the act of the plaintiff, or by his physical debility, or his scrofulous habit, or his restlessness or his tossing about, or his cough, or by any accidental cause, the defendants would not be liable for any damages resulting from such causes. And if the jury believe, from the evidence, that the leg of the plaintiff was left in an unusually imperfect state, when under the treatment of the defendants, still they cannot find the defendants guilty, unless they believe, from the evidence, that such imperfections did not result from any of the causes above mentioned.

6. A surgeon, when called upon to treat a fractured limb, is not bound to attend all the time in person upon his patient; but he is bound only to call upon his patient at such reasonable times as are customary and reasonably proper among surgeons in such cases.

7. Where a surgeon is employed to treat a fractured limb it is not implied on his part to make a perfect limb, from the fact of such employment, but he must use reasonable skill and give reasonable attention to bring about such a result; and if, after giving such attention and skill, he fails to accomplish it, he is not responsible.

8. In this case the opinion of competent surgeons and physicians as to the skillfulness of the manner of the treatment of the plaintiff's leg, and also as to whether the plaintiff's leg after such treatment was as reasonably good as could have been expected, are to be received by the jury as proper evidence to prove or tending to prove such facts.

Verdict for defendants.

The Statute of Limitations for Illinois requires that "actions for damages for an injury to the person shall be commenced within two years next after the causes of action accrued." *Gross' Statutes.*

I cite twenty-three cases, with causes of action and verdicts. Surgeons will see from these cases the most they have to fear is the cost of their defense.

*Howard* v. *Grover*, 28 *Maine R.* 97; amputation. The surgeon, for error of judgment, was mulcted in $1,500 damages. *Slater* v. *Baker*, 2 *Wilson*, 250; refracturing a broken leg without consulting patient's wishes. Damages against surgeon, $2,500. *Gallaher* v. *Thompson*, *Wright's Ohio S. C. R.* 466; fractured leg. Verdict for the defendant. *McCandless* v. *McWha*, 22 *Penn. R.* 261. *Livingston Law Mag.*, *June*, 1855; fractured leg. Verdict in lower court against the surgeon, for $850. Judgment reversed, and sent back for a new trial. *Leighton* v. *Sargeant*, 7 *Foster*, 460. Verdict for plaintiff, $1,500. Judgment reversed. The above five cases are cited in *Elwell's Malpractice.*

*Landon* v. *Humphrey*, 9 *Conn.* 209; vaccination. Verdict against surgeon. *Long* v. *Morrison*, 14 *Ind.* 595; patient died under treatment; disease not given. Against surgeon. *Peck* v. *Martin*, 17 *Ind.* 115; child under treatment, became insane. Verdict against surgeon. *Cochran* v. *Miller*, 13 *Iowa*, 128. Case went up on admission of testimony and instructions to jury. Nature of the case not given. Verdict against the surgeon. *McMillen* v. *Hewett*, *Sprague* and *Rodman*, *Elwell's Malpractice;* iritis. For the surgeon. *Holt* v. *Breck*, *Elwell's Malpractice;* incised wound. Never came to trial. *Grinnell* v. *Riggs;* false joint in fractured arm. For surgeon. *Med. and Surg. Report, July* 20, 1861. *Gattie* v. *Halford*, *London C. of Exch.;* using instruments in labor, without assistance. For the surgeon. *M. & S. Reporter, Aug. 3rd*, 1861. *Russell* v. *Wardner;* broken fore-arm. For defendant. *Ibid, May* 20, 1871. *Ordway* v. *Haynes;* fractured thigh. For the surgeon. *Ibid.* Of five cases, all in Middlesex Co., Mass., none of the plaintiffs received a single cent. In one case a verdict was obtained against the surgeon on the testimony of a "natural bonesetter," the judge being a warm admirer of said "bonesetter." *Welch* v. *Sayre;* scrofulous abscess of hip. Plaintiff averred that Dr. Sayre had punctured the hip joint in opening the abscess. Verdict for the surgeon. *N. Y. Med. Jour., vol. xi*, 581. *Haire* v. *Reese;* simple contusion of the hip, followed by "interstitial absorption of the neck of the thigh bone." For the surgeon. *Ibid, vol. xiii*, 124; *Med. Times, Dec. 1st*, 1870. *Fisher* v. *Gross;* secondary hemorrhage. For the surgeon. An attorney was found in this last case who agreed to pay all expenses and to give his services beside, the consideration being a certain per centage of the damages. *Med. Record, vol. vi*, 133.

The union of "*shyster*" and "*quack*," are frequently met with in suits for malpractice.

### NEGLIGENCE AND SKILL FROM A MEDICAL STAND POINT.

We shall limit ourselves, in discussing the matter of skill and negligence, from the medical stand-point, to the consideration of fractures, dislocations and amputations, these forming the larger number of the cases that come into courts for adjudication. What do our eminent teachers say in regard to these questions ?

DIAGNOSIS.—The first duty the surgeon has to perform, when called in case of accident, is to determine the nature of the injury. Is it a bruise, a sprain, a dislocation, or a fracture? In injuries near joints, the diagnosis will be, often, most difficult to make. The rapid effusion and consequent swelling that takes place, will tax the surgeon's skill to the utmost, especially when the injury is near the shoulder, the elbow or the hip joints. The points claiming the particular attention of the surgeon relate to deformity, shortening or lengthening, preternatural mobility, or immobility and crepitus. The recognition of deformity will be made out by the eye taking the uninjured limb for comparison. It may be necessary to use the "touch" as an aid to the sight, but in this as in all other manipulations the surgeon should exercise the greatest gentleness. It is no mark of surgical attainment to handle a broken limb roughly, neither is it necessary, and the surgeon who does so, could be easily held to a charge of unskillfulness. *Hamilton Fract. and Dis.*, *3d ed.*, *p.* 34, *et sequentes.* The statement above should be qualified by "*as a rule*," for there, undoubtedly, are cases in which, for the best interest of the patient, the surgeon would be justified in manipulating the injured limb throughly and for some length of time, indeed he would be obliged to, before he could come to anything like a correct conclusion in the case. *Gross' System of Surg.*, *Vol.* 1, 3*d ed.*, *p.* 860.

Difference in the relative length of the limbs is to be determined by careful measurement. In fractures of the femur, measurement should always be practiced, not once only, but every day, or as frequently as the surgeon may deem it necessary, in order to determine that his dressing is fulfilling its purpose in keeping the broken bone to as near its proper length as the nature of the case will permit.

Preternatural mobility is a sign, present in all fractures, *except impacted*, and should be carefully looked for by the surgeon. Not so important as crepitus, yet being always present it is scarcely less important.

Crepitus, that grating sound *heard* not only by the ear but also *felt* by the hand of the surgeon, is the most valuable of the common signs, but unfortunately it is not always present. The ends of the broken bones may be impacted, as often happens, near the joints, which, with the great amount of swelling, so obscures the nature of the accident that eminent surgeons have been frequently led into error.

Prof. Hamilton, (*Fract. and Dis.*, *3d ed.*, 34,) speaking of general diagnosis says: "Valuable and important as is crepitus in its relations to differential diagnosis, unfortunately it is not always present, and for reasons that must be plainly stated. First: we cannot, in a pretty large proportion of cases, bring the broken ends again into apposition. Whatever mere theorists may say to the contrary, and notwithstanding surgeons up to this time have rarely ventured to allude to this subject, the fact is so that we do not "set" broken bones. We do not, even at first, bring them into complete apposition, unless it is the exception. I speak of bones once completely displaced by overlapping, and these constitute the majority of examples which come under the surgeon's observation. Second: in transverse fractures of the patella, and in fractures of the olecranon and coronoid processes of the ulna, of the coracoid and acromion process of the scapula, and in all similar detachments of processes and apophyses, the action of the muscles, by displacing the fragments, prevents crepitus from being readily produced. Third: in a few cases, such as certain fractures of the neck of the femur, of the neck and head of the humerus, etc., the broken ends are impacted, or so driven into each other as to forbid the production of motion and crepitus; or they may be simply denticulated, and the consequences, so far as crepitus is concerned, will be the same."

We may remark here, that the diagnosis of dislocations is often made with the greatest difficulty, and may not be made correctly at all, from the great amount of swelling that almost instantly supervenes. Sir Astley Cooper remarks, (*Dis. and Frac.*, *Am. ed.*, 319,) when speaking of the signs of dislocations of the humerus—and the remarks are equally applicable to fractures of the humerus near either end—that, "but a few hours make the appearances much less decisive, from the extravasation of blood, and from the excessive swelling, which sometimes ensue; but when the effused blood has become absorbed, and the inflammation has subsided, the marks of the injury became again decisive. At this latter period it is that surgeons of the metropolis are usually consulted; and if we detect a dislocation which has been overlooked, it is our duty, in all candor, to state to the patient that the difficulty of detecting the nature of the accident is exceedingly diminished by the cessation of inflammation and the absence of tumefaction. It may also be observed that there is great difference in the facility with which the accident is discovered in thin persons of advanced age, and in those who are loaded with fat, or who have, by constant exertion, rendered their muscles excessively large." He remarks, previously, in his general observations on dislocations, that "it must be borne in mind that where fibrinous effusion has taken place, as generally happens in inflamed joints, we may have sounds that no skill can determine the nature of, especially in such cases as above described." See also, *Ashurst Princip. and Prac. of Surgery*, 270–1.

The chafing sound, which is frequently present in dislocations and inflamed joints, resembling the crepitus of fractures, will often lead the inexperienced, and sometimes the experienced, into error. If, however, the surgeon applies his differential tests carefully, he should not and would not be held liable to damages for such errors of judgment.

Dislocations, as a rule, are characterized by preternatural immobility, and when reduced do not need support to retain the bone in position. Observation of the above characteristics will carry the surgeon safely through, unless there be both fracture and dislocation, in which case the difficulty in diagnosis will be most annoying.

The following case illustrates these difficulties well. By the kindness of the patient, I am able to present him for examination at this meeting.

W. J. Adams, dentist, age 25, was thrown from a horse, Nov. 21st, 1872. Saw him within three-quarters of an hour. The force of the fall was received on the left forearm and arm. The chief injury was located in and around the elbow-joint. Found him with the forearm flexed at a right angle; elbow very much swollen; no particular difficulty in extending the arm or flexing it; pronation and supination not more impaired than they would be from pain and the swelling of the muscular tissues. The patient was quite faint from shock; rotation of head of radius could not be felt on account of tumefaction; in extended position, rotation developed crepitus. Passing fingers up the ulna discovered a depression about an inch and a quarter to an inch and three-quarters below point of olecranon; flexion of the arm increased this depression somewhat; there was a sharp projecting edge on upper fragment, which was quite prominent when forearm was flexed. Diagnosed fracture of the olecranon process at its base.

Never having seen a fracture of this part, in my own experience or the experience of others, and carrying in my mind that there was a difference of opinion as to the position in which the arm should be dressed, I went to my office to prepare a splint and consult my authorities on the subject. The weight of authority being in favor of the straight position, it was so dressed, on a long tin gutter splint applied to front of the arm, secured by a roller bandage figure of eight around the joint. This dressing was continued till the afternoon of the 23rd, when, at patient's request, it was left off for a few hours. The patient had found it necessary, on account of the swelling and pain, to cut the figure-of-eight bandage several times since first dressing. The arm was dressed twice a day for the first three or four days. While the splint was off, had him apply water dressing, of such temperature as was most agreeable, arm being kept in extended position. Had the arm wet frequently since the first dressing with camphor spirits, tinct. arnica, etc. The swelling commenced subsiding by the 24th, the pain being much less. There was by this time a very great amount of ecchymosis developed upon the inner and upper portion of the arm as high as the axilla. I had told patient that at the end of third week I would put in practice passive motion. Patient went to another county the third week (20th day) of the treatment, and did not return for about ten days. During his absence several surgeons saw the arm and thought it was doing well. When I next saw him, the swelling had in a great measure subsided; there was some ability to flex and rotate the arm by means of the other hand. There was, at this time, and there continues to be, that grating sound, resembling crepitus, which I refer to inflammatory exudations. The fragments appear to have become united with but little separation. There is a slight excess of projection in the vicinity of the outer condyle or perhaps as low as the head of the radius. This, doubtless, is the displaced fractured external condyle. The depression at seat of fracture is easily recognized; both flexion and rotation under passive and active exercise seems to be improving.

The difficulties here presented were—1st. Danger of separation of fragments. This I prevented by dressing the arm in the straight position. 2. The fracture being so low down, the joint might be implicated, and there would be permanent anchylosis from osseous deposit in the joint. 3. Fracture so low down that head of radius would be intruded upon by callus, and so rotation impaired. 4. Dislocation of head of radius backwards or outwards, or fracture of the same. 5. Fracture of external condyle. As will be seen from history of the case, the backward dislocation was eliminated by the ability to flex and extend the forearm easily, also the ability to rotate forearm. An outer dislocation was eliminated by same tests; fracture of condyle not evident, on account of swelling; and all were ignored from a general absence of deformity. The crepitus of fracture was referred entirely to the olecranon process.*

From all this, we may, therefore, conclude, that, as " diagnosis is a science of probabilities," the surgeon who faithfully considers these tests, and promptly applies them to the elucidation of his case, has skillfully performed his duty in diagnosis.

SKILL.—What constitutes skill in treatment? The indications are, first, to put the bones in as near apposition as possible; secondly, to keep them in this position; thirdly, keep down or allay spasm and undue inflammation; and fourthly, the management of the accidents that may occur during treatment. The first indication is accomplished by gentle extension and counter-

* The joint was critically examined by Dr. Hamilton, of Galesburg, who thinks there was probable fracture of external condyle, with fracture of olecranon. In this opinion, Drs. Reece, Nance, Phillips, Aldrich, Purdum, Webster, Hurd, Babcock, Maxwell, Nelson, McCutchan and other gentlemen of the society concurred.

Drs. Hamilton and Reece favored the use of an anæsthetic and a judicious amount of force to bend elbow at right angle.

All the other gentlemen advised severe passive motion, either with or without an angular-jointed splint, with thumb-screw to procure gradual flexion.

extension, with pressure at the seat of the fracture; the second by splints, bandages, and a continuance of the extending and counter-extending force; the third by anodynes, water-dressings, etc. Let me call the especial attention of the surgeon to the danger of using the primary or initial bandage. Notice how many cases come into courts, in which this is one of the apparent causes of trouble. The older surgeons were in the habit of applying this dressing to a broken limb before applying the lateral splints. The object to be obtained by it, *i. e.*, support of the muscles and thus prevention of their contraction, and the protection of the limb from direct contact with the sides of the splints, are desirable. The same objects can be accomplished, as well, by properly padding the splints, and by the use of the roller bandage in retaining them. The danger of so ligating the limb as to occasion congestion, inflammation and gangrene, is so great, by such dressing, that the surgeon would hardly be held "not guilty," who should use it. I would here, therefore, enter my earnest protest against the use of this dressing. In my first case of fracture of the leg, another surgeon, presuming on his *seniority* in practice, applied such a dressing, contrary to my objections, the sequence of which was a narrow escape from a suit for malpractice. The writer was exonerated from blame in the case inasmuch as he had two *intelligent friends*, who were witnesses to the protest.

Dr. Ashurst remarks (*Princip. and Prac. of Surg.* 223–6): "CIRCULAR COMPRESSION is to be carefully avoided, as swelling is inevitable after a fracture, and the risk of gangrene from this cause is by no means only theoretical. Hence, as a rule, in the early stages of fractures, *no bandages should be applied beneath the splints.* Gangrene is the most serious accident which can be met with in the treatment of a simple fracture, and may be due either to arterial obstruction at a point above the seat of fracture, to venous obstruction due to swelling of the part, or to too tight bandaging, or to a combination of these causes. With regard to tight bandaging it is to be remembered that a bandage may be sufficiently loose when applied, and yet in a few hours may become the cause of great constriction from subsequent swelling of the limb; hence the importance of not *applying a bandage beneath the splints;* it is, as remarked by Mr. Ericksen, almost invariably to a neglect of this rule that the occurrence of gangrene from the pressure of a bandage is due. Especially is this true in the case of the forearm, in fractures of which part this accident most often occurs. It should not be forgotten, however, that this accident may be partly or entirely due to arterial obstruction, which is, of course, an unavoidable occurrence; hence we should not be too hasty in accusing a fellow-practitioner of malpractice on account of such an accident, for it may be really due, at least in some measure, to causes entirely beyond control. The *treatment* of gangrene occurring under such circumstances must vary according to its nature and extent; if it be due to constriction, and the surgeon fortunately discovers it in time, he must instantly remove the bandages, when possibly the patient may escape with superficial sloughing. If complete gangrene has occurred, amputation of course becomes necessary; if the disease show a disposition to self-limitation, the surgeon may await the formation of the lines of demarcation and separation, but if the gangrene be of the rapidly spreading traumatic variety, immediate removal of the limb must be practiced at a point above the furthest limits of the disease."

Dr. Ashurst, in speaking of the reduction or "setting" of fractures, says that "it consists in replacing the fragments by manipulation as nearly as possible in their normal position as regards each other. I say advisedly, 'as nearly as possible,' for I believe with Prof. Hamilton, that it is only in exceptional cases that the displacement of fractures can be entirely overcome."

I have been induced to quote thus largely from Ashurst because he is one of the latest writers on the subject, and stands high as a teacher and practitioner. To him and to Prof. Hamilton, the profession owes a debt of gratitude for the *honest* statements they have made, in respect to the conditions that obtain in the matter of fractures.

The next question that claims our consideration is, when should a fracture be reduced—immediately, or not till the inflammatory action consequent upon the injury has been subdued? "No greater absurdity and cruelty are conceivable than leaving the fracture unadjusted." *Liston's Elements of Surg., Am. ed.*, 581. "Five good reasons why broken bones should be reduced as soon as possible: 1, when the injury is recent, the muscles offer less resistance; 2, their resistance is increased not only by reaction but also by actual adhesions between their fibers; 3, effusion distends both the muscles and the skin, and compels the limb to shorten; 4, the constant goading of the flesh by the sharp points of the broken bones increases the muscular contractions; 5, the patient will submit readily to manipulation and extension at first, but after the lapse of a few days it is very seldom that he will permit the limb to be in any manner disturbed, even if he is assured that his refusal entails upon him a great deformity." *Hamilton Frac. and Dis.*, *3d ed.*, 44, *et seq.* "It appears singular that upon a subject so clear as this there should be any difference of opinion. It certainly requires no great knowledge of the nature of accidents to discover why such cases should receive the earliest possible attention: as long as the ends of the fragments are permitted to remain apart, their tendency inevitably must be to excite spasm and inflammation, thereby increasing the suffering of the patient and retarding his cure." *Gross, vol.* 1, 865. "As a broken bone is a constant source of irritation to surrounding parts, and the periosteum is liable to be separated to a greater extent in proportion to the spasmodic motion of the muscles, it is singular that any doubt could exist as to the advantage of setting a fracture as soon as possible." *H. H. Smith, Op. Surg., vol.* 1, 540. "Reduction should be effected *as soon as possible*, for the reason that it is much easier for the surgeon and much less painful to the patient, if done before the development of inflammation; if, however, the patient, is not seen until a later period, or if displacement should, from any cause, have recurred, the surgeon need not hesitate at any stage of the case to effect as perfect a reduction as he can, for the slight additional irritation thus produced will be of much less consequence than the evils which would result from continued displacement." *Ashurst Princip. and Prac. Surg.*

Failure to reduce a fracture promptly would constitute negligence, which we shall consider more in detail, when we come to treat of the accidents liable to occur during the cure of a fracture.

(To be continued.)

